# Quality Characteristics of Twelve Advanced Lines of *Avena magna* ssp. *domestica* Grown in Three Contrasting Locations in Morocco

**DOI:** 10.3390/plants13020294

**Published:** 2024-01-18

**Authors:** El hadji Thiam, Michael Dunn, Eric W. Jackson, Eric N. Jellen, Mark Nelson, Will Rogers, Carol Wallace, Gene Ahlborn, Majid Mounir, Teresa Yakovac, Shane Morris, Ouafae Benlhabib

**Affiliations:** 1 Plant, Production, Protection and Biotechnology Department, Institut Agronomique et Vétérinaire Hassan II, Rabat 10000, Morocco; elasthiam0@gmail.com; 2 Nutrition, Dietetics and Food Science Department, Brigham Young University, Provo, UT 84602, USA; michael_dunn@byu.edu (M.D.); gene_ahlborn@byu.edu (G.A.); 3 25:2 Solutions LLC, 815 S First Ave Suite A, Pocatello, ID 83201, USA; eric.jackson@25-2.com (E.W.J.); teresa.yakovac@25-2.com (T.Y.); shane.morris@25-2.com (S.M.); 4 Plant and Wildlife Sciences Department, Brigham Young University, Provo, UT 84602, USA; jellen@byu.edu; 5 Resourced Inc., 304 East Main Street #148, Mahomet, IL 61853, USA; mark.nelson@resourced.global (M.N.); will.rogers@resourced.global (W.R.);; 6 Food Science and Nutrition Department, Institut Agronomique et Vétérinaire Hassan II, Rabat 10000, Morocco; m.mounir@iav.ac.ma

**Keywords:** *Avena magna* ssp. *domestica*, advanced lines, oat groats, chemical composition, macronutrients, protein, minerals, zinc, iron, β-glucan, correlations, stability

## Abstract

The popularity of oats (*Avena sativa*) continues to increase in the cereal market due to their health benefits. The recent domestication of *Avena magna*, a Moroccan oat, presents an opportunity to enhance these benefits due to their higher nutritional composition. As the impact of microclimates on *A. magna* grain composition has not been explored, this study evaluates twelve *A. magna* ssp. *domestica* lines across three Moroccan locations, providing new data into microclimate effects on key grain characteristics. Significant variability is observed among lines and sites for nutrients, with mean protein, fat, and dietary fiber contents at 23.1%, 8.38%, and 7.23%, respectively. High protein levels, reaching 27.1% in Alnif and 26.5% in El Kbab, surpass the ‘Avery’ control (21.7% and 24.2%) in these environments. Groats from Bouchane exhibited elevated fat and fiber contents (10.2% and 9.94%) compared to the control (8.83% and 7.36%). While β-glucan levels remain consistent at 2.53%, a negative correlation between protein content, fat, and starch was observed. *A. magna* lines exhibited higher levels of iron (7.50 × 10^−3^ g/100 g DM) and zinc (3.40 × 10^−3^ g/100 g DM) compared to other cereals. Environmental conditions significantly influence grain quality, with El Kbab yielding higher protein and ash contents, as well as Bouchane having increased fat, fiber, and starch. Stability analysis indicates that fat content was more influenced by the environment, while 25% of protein variability is influenced by genetics. Lines AT3, AT5, AT6, AT13, and AT15 consistently exceeds both the mean for protein and fiber across all sites, emphasizing their potential nutritional value. This study highlights the potential of *A. magna* ssp. *domestica* to address nutritional insecurity, particularly for protein, iron, and zinc in domestic settings.

## 1. Introduction

The world’s population is growing rapidly and is expected to exceed 9 billion by 2050 [1]. This rapid population growth is putting pressure on resources and will increase global food demand by 50–60% between 2019 and 2050 [2]. At the same time, climate change and the reduction of arable land due to urbanization are significantly affecting crop production [3,4,5]. In addition, more than 820 million people are chronically undernourished in terms of energy, and 2 billion people are deficient in micronutrients such as iron and zinc [6,7]. Access to nutritious and sustainable food, both presently and in the future, is a primary concern and will be critical in alleviating both chronic and hidden hunger.

In this context, cereal breeders are beginning to focus on traits related not only to yield, but also to nutrition and health, as studies demonstrate the impact of cereal consumption on human health emerge [8]. Increased consumption of whole cereal grains has been shown to play a key role in reducing obesity, type II diabetes, gastrointestinal disorders, as well as coronary heart disease and certain types of cancer [9,10]. One cereal crop that is receiving significant attention in this regard is *Avena sativa*, the common oat, which has attracted the attention of researchers and growers because of its potential health benefits [11]. Oat species are known to be good sources of β-glucan, a soluble dietary fiber that plays a prominent role in reducing serum cholesterol and glucose uptake, decreasing blood insulin response, controlling weight by prolonging satiety, and balancing the gastrointestinal function in humans [12]. Among staple grains, oats also have the highest protein content [13]. In addition, oats are gluten-free and are tolerated by most people with celiac disease [14,15]. Agronomically, oats are fairly hardy, and can grow in a variety of climates and environments, even showing tolerance to wet weather and acidic soils. They require less use of agrochemicals and fertilizers, so their production costs can be lower than those of wheat or barley [16]. There are several different species of oats in production, with the common spring or white oat, *A*. *sativa* (2n = 6x = 42, AACCDD), being the most important cultivated form. *A*. *byzantina* (2n = 6x = 42) is a red oat species adapted to warmer climates where it is grown as a winter oat. Because of its greater heat tolerance, *A*. *byzantina* was used as a parent with *A. sativa* in the development of one of the first oat varieties bred in Morocco, “Tutrice 153”, in 1924 [17].

In the search for even greater opportunities for cereal improvement, many plant scientists are looking to crop wild relatives as important sources of desirable genes that could improve nutrition, mitigate the effects of climate change, and help feed the growing population [18,19,20]. Neo-domestication is an important means of diversifying the existing cultivated crop germplasm [21]. In fact, the current limited agricultural diversity is very risky and has serious consequences for food security and global biodiversity [22]. The main source of diversity for the improvement of cultivated oats has been the genetic pool residing in oat wild relatives [23]. Many previous studies have attempted to transfer the high protein content of some wild relatives to the cultivated *A*. *sativa*, with *A*. *sterilis* being the most commonly used. However, in some cases, the higher protein content found in the resulting progeny was accompanied by undesirable traits such as increased husk content in the grain, as well as a reduction in grain filling, reduced yield, spikelet shattering, and awn formation [24].

A more recent example of a wild relative making inroads into oat breeding circles is another oat of Moroccan origin, *A*. *magna* (2n = 4x = 28, CCDD genome), a wild tetraploid that has attracted increasing interest due to its unique grain composition. It is reported to have a higher protein content than common oats—among the highest of the true cereals, at 25–30%—and is a good source of iron and zinc [25,26]. Increased dietary intake of these essential nutrients could benefit the 1.5 billion people worldwide who are deficient in iron and zinc in their diets [27].

Because of the potential nutritional benefits of *A*. *magna*, its agronomic domestication has been carefully studied and developed over the past 25 years. The newly domesticated Moroccan tetraploid species *A*. *magna domestica* (2n = 4x = 28, CCDD genome) was developed via sexual transfer of the domestication syndrome from the hexaploid common oat *A*. *sativa* (2n = 6x = 42, AACCDD; [25]). Subsequently, several lines were further developed by mutagenesis [26]. Some of these lines have recently demonstrated their suitability for large-scale production in their native Morocco [28,29]. However, studies on the chemical composition of the most recently developed germplasm grown in Morocco are lacking. Most published compositional data have focused on the nutritional evaluation of earlier *A*. *magna* ssp. *domestica* breeding lines [26,30]. In addition, the potential influence of microclimate on *A*. *magna* grain composition in the different growing regions of Morocco has not been investigated.

There is a critical need to characterize the chemical composition of the latest *A*. *magna* ssp. *domestica* lines in parallel with ongoing conventional breeding efforts, to identify cultivated germplasm that retains the nutritional health benefits of the wild *A*. *magna* parents. At the same time, exploring the relationship between genotype, the growing environment, and key quality traits will be useful for further selection. As a contribution to this endeavor, the present work aims to assess the chemical composition of twelve advanced lines of *A*. *magna* ssp. *domestica* grown in three different locations in Morocco and to shed light on how some of their nutritional components respond to the genotype × environment interaction (G × E).

## 2. Material and Methods

### 2.1. Plant Materials

Eleven *A*. *magna* ssp. *domestica* advanced breeding lines (AT1, AT2, AT3, AT4, AT5, AT6, AT7, AT9, AT13, AT14, and AT15) and the ‘Avery’ control (ATC) used in this study were selected from a set of 41 *A*. *magna* ssp. *domestica* breeding lines provided by General Mills, Inc. in Minneapolis, MN, USA. The eleven lines were selected based on yield, disease tolerance, production stability, and lemma pubescence (*Lp*) following adaptation trials over two growing seasons in Morocco, 2018 and 2019 [28]. Seeds for the nutritional analysis were collected from the 2020–2021 trials in Alnif, Bouchane, and El Kbab, Morocco. The location and the growing conditions are shown in Table 1. After harvest, the oat grains were dried at 35 °C and stored below 18 °C at the Institut Agronomique et Vétérinaire Hassan II (Rabat, Morocco) until use.

### 2.2. Chemical Analysis

Chemical quality analyses were conducted during the summer of 2022 at the 25:2 Solutions CIPHER Lab, Pocatello, ID, USA; Brigham Young University, Environmental Analytical Lab (EAL), Provo, UT, USA; and Analytical Food Laboratories (AFL), Grand Prairie, TX, USA. Grains were first dehulled using the Codema LH 5095 Laboratory Oat Huller (Codema, Lublin, Poland), then ground and sieved using the UDY Corporation Cyclone Lab Sample Mill (Fort Collins, CO, USA). The collected flour samples were stored for subsequent analyses. Moisture, fiber, ash, and starch were determined using a near infrared spectrometer (FOSS NIRS™ DS2500 F (Hilerod, Denmark)) in the 25:2 CIPHER Lab. Protein and fat were determined in the 25:2 CIPHER Lab using the LECO Crude Protein Method (LECO, St. Joseph, MI, USA) according to AOAC Official Method 990.3 [31] and AOAC Lipid Extraction Acid Hydrolysis Method accordingly to AOAC Official Method 922.06 [32], respectively.

Mineral content was determined by inductively coupled plasma (ICP) spectroscopy at EAL, according to the AACC 40-75.01 [33], where seed samples are burned in a muffle furnace to collect the ash residue. After dissolution in dilute acid, the ash mineral concentration of each sample was quantified using ICP spectroscopy measurements.

Measurements of β-glucan content were performed using the MEGAZYME^©^ Mixed-Linkage β-Glucan Assay Procedure (AOAC Method 995.16) [34]. All the analyses were performed in duplicate. Values are expressed as g/100 g dry matter (DM) for macro and micronutrients.

### 2.3. Statistical Analysis

The chemical analysis data were subjected to the R statistical software version 4.2.1 [35] to assess the variability among lines and experimental sites. Factominer version 2.4 with Factoshiny [36] and Agricolae packages were used for the principal component analysis (PCA), dendrogram, and AMMI analysis, respectively. Pearson’s correlation was used to study the relationships between the chemical components. Two-way analysis of variance (ANOVA 2) with Newman–Keuls honestly significant difference post hoc was used to determine whether means of *A*. *magna* lines and growing sites were significantly different at 95% level of confidence (*p* < 0.05). Values were expressed as the mean ± standard deviation. The AMMI Stability Value (ASV) based on the AMMI model is calculated for protein, fat, and fiber according to the formula developed by Purchase [37].

## 3. Results and Discussion

### 3.1. Chemical Composition

Macronutrient content, ash, and moisture of the *A*. *magna* oat lines at different growing locations are shown in Table 2.

According to the ANOVA, there were significant differences in composition between sites and lines for all components, except β-glucan. These results suggest that the chemical composition of these *A*. *magna* lines is influenced by both genetic factors and the growing conditions, as has been reported in many previous studies on cereals [16,38].

The moisture content ranged from 12.6 to 12.9%, which was higher than the moisture values reported by Manzali et al. [30] for *A*. *magna* groats, but quite similar to the typical moisture of other common cereals, including *A*. *sativa*, as reported in Table 3. Nelson et al. [39] mentioned that the time of harvest and storage conditions affect the moisture content of cereal grains, so differences in moisture from lot to lot are not unexpected. Moisture content of cereal grains is an important quality characteristic as it can dramatically affect shelf life and is considered in determining the end use of the product. The samples used in this study were dried after harvest and stored for six months prior to analysis. It is possible that storage and processing at ambient conditions during dehulling increased moisture absorbance.

Considering the sites separately, Table 2 shows that the average protein content varied from 20.9% in Bouchane, on the Phosphate Plateau, to 24.1% in the irrigated oasis site of Alnif. The highest protein contents in Alnif were observed in lines AT3, AT6 (both at 27.1%), and AT5 (27.0%). The site with the next highest protein content was the mountainous site of El Kbab, where lines AT6, AT15, and AT13 yielded 26.5, 25.8, and 25.8% protein, respectively. The Bouchane site exhibited the highest fat and fiber contents among the individual lines, with AT14 (10.2%), AT7 (10.2%), and AT15 (10.1%) having the highest percentage of fat, and AT13 (9.94%), AT3, and AT6 (both at 9.23%) having the highest amount of fiber.

With respect to the individual lines across all sites, seven lines had significantly higher protein content than the Avery control (Table 2, ATC). AT6 had the highest protein content overall (25.5%); its protein values in each environment were also directionally higher as well, with 22.9, 26.5, and 27.1% in Bouchane, El Kbab, and Alnif, respectively. Furthermore, the protein content of the current advanced lines in this study is higher than that reported by Manzali et al. [30] for nine *A*. *magna domestica* lines and by Saidi et al. [41] for eight hexaploid recombinant inbred lines (RILs) developed between two wild *A*. *magna* accessions and five local *A*. *sativa* cultivars. The mean protein content of the Avery control (22.4%) was lower than the values of >25% reported by Jackson [26], but the Avery protein in El Kbab was close (24.2%), highlighting the environmental influence on this trait. Ladizinsky [42] reported a range of 23–27% for *A*. *magna* wild types in his study. AT5 and AT6 provided 27.0% and 27.1% of the protein content in Alnif, respectively, confirming the conservation of this trait during the domestication process of the studied lines. The differences in protein content were undoubtedly influenced by one or more factors, including the geographical location, cultivation and storage conditions, and technical analysis. It should also be noted that the *A*. *magna* strains in our study were derived from a cross between one of Ladizinsky’s original 28-chromosome *A*. *magna* × *A*. *sativa* backcross lines and a wild *A*. *magna* to produce a segregating population from which lines were selected at various generations of inbreeding [26,28].

The mean fat content ranged from 7.92% for AT4 to 8.78% for AT14 (Table 2). Among the samples, the minimum and maximum values were 6.44% for AT4 in Alnif and 10.2% for AT14 in Bouchane, respectively. The lipid values obtained for these advanced lines are typical of those reported elsewhere for *A*. *magna* [30] and *A*. *sativa* [15], but exceed those for common wheat, barley, maize, rice, and millet in Table 3 [40]. In general, oats have the highest fat content among cereal grains. In this study, there was a significant negative correlation between protein and fat content (−0.764).

For fiber content, the minimum and maximum values between samples were 5.77% for AT4 in El Kbab and 9.94% for AT13 in Bouchane, respectively. All advanced lines except AT1 had values above ATC, with six of them being significantly higher (Table 2). The fiber value of AT6 was the highest (8.00%), significantly higher than the control and the other lines except AT13 (7.96%). The ash content varied in a narrow range from 2.42% (AT14) to 2.72% (AT3), which is consistent with the literature data for *A*. *sativa* oats [15,43]. The ash content of AT3 was significantly higher than that of the control and five of the advanced lines (AT2, AT4, AT7, AT9, and AT14).

Starch is the main storage carbohydrate in cereals and its content in oats can vary from 39 up to 70% depending on the species and the cultivar [40,44]. The starch content of the advanced lines ranged from 54.9% for AT6 to 58.1% for AT7.

β-glucan is a non-starch polysaccharide composed of linear D-glucose chains bound by mixed β-(1,3)- and β-(1,4)-glycosidic linkages. Soluble dietary fiber has been associated with a reduced risk of cardiovascular disease, type II diabetes, and gastrointestinal cancer [45,46]. β-glucan levels did not differ significantly between lines or sites. The results for the *A*. *magna* advanced lines in this study were lower than those reported in the literature for *A*. *sativa* (3.9–5.7%) [47]. However, they were consistent with those reported for *A*. *magna* reported by Jackson [26]. Despite having lower β-glucan levels (a 2.53% mean) than *A*. *sativa*, the levels of the advanced lines are still higher than the other common cereals, with the exception of barley [40,48]. In addition, oats are also rich in other healthy dietary fibers such as arabinoxylans [49]. Therefore, it is worth investigating whether this type of healthy fiber may compensate for the low β-glucan content in some *A*. *magna* lines. These results indicate that, from a compositional point of view, protein partially replaces fat and, especially, fiber in the *A*. *magna* lines.

In addition to the β-glucan levels discussed above, Table 3 illustrates the balanced chemical composition of the advanced *A*. *magna* lines, compared to other common cereals. They are higher in protein (23.1%) and, compared to *A*. *sativa*, still retain a reasonable amount of ash/minerals and lipids (8.38%), the latter of which, as is typical for cereals, are predominantly the healthier mono- and polyunsaturated varieties [26].

Since the advanced lines were grown in different locations, the macronutrient results are not only due to genetic factors but also to the environment and its interaction (G × E). According to Redaelli et al. [50], starch accumulation is more sensitive to high temperatures than nitrogen accumulation, which often determines seed protein content. This could explain the higher starch loading in the drier environment of Bouchane (57.7%) compared to Alnif (57.2%) and El Kbab (56.9%), the less water restrictive environments. On the other hand, the protein content was higher in El Kbab (24.2%) and Alnif (24.1%) compared to that in Bouchane (20.9%).

### 3.2. Mineral Composition

Essential minerals are very important in the human diet. Therefore, increasing the accumulation of minerals in cereal grains is one of the sustainable strategies used by plant breeders to help combat malnutrition [51]. The statistical data analysis of the present investigation indicates that the mineral composition of *A*. *magna* differs significantly, and sometimes substantially, among lines and sites for most of the minerals tested (Table 4). Of particular note was the advanced line AT2, which had directionally the highest values of calcium (0.77 × 10^−1^ g/100 g DM), iron (9.33 × 10^−3^ g/100 g DM) and copper (0.59 × 10^−3^ g/100 g DM), while AT13 provided the highest values of zinc (3.70 × 10^−3^ g/100 g DM). Five lines (ATC, AT3, AT6, AT14, and AT15) were directionally higher than the others in average potassium content, ranging from 3.62 × 10^−1^–3.68 × 10^−1^ g/100 g DM, but only AT5 was significantly lower. Copper ranged from 0.49 × 10^−3^ to 0.59 × 10^−3^ g/100 g DM for AT4 and AT2, respectively, while manganese varied from 4.23 × 10^−3^ to 4.90 × 10^−3^ g/100 g DM. Calcium content of AT14 was directionally the lowest among the advanced lines, with significant differences from AT2, AT6, and AT13, but the mean calcium content for all treatments (0.70 × 10^−1^ g/100 g DM, as is) was almost 20% higher than the value of 0.51 × 10^−1^ g/100 g DM reported by Jackson [26] in previous breeding lines of *A*. *magna* and higher than all other common cereals (see Table 5).

The advanced lines also showed their micronutrient richness in iron, zinc, and phosphorus contents compared to other cereals (Table 5). The mean iron content of 7.50 × 10^−3^ g/100 g DM (as is) is higher than that of *A*. *sativa*, or any of the other common cereals. The average zinc content of 3.40 × 10^−3^ g/100 g DM (as is) and phosphorus content of 4.57 × 10^−1^ g/100 g DM (as is) of the advanced lines studied in this investigation exceed the values reported for the other cereals in Table 5, although they are lower than those of *A*. *sativa*. Although there are no significant differences between lines, the iron level of AT2 (9.33 × 10^−3^ g/100 g DM) is similar to the value of 9.10 × 10^−3^ g/100 g DM reported by Jackson [26]. It is worth noting that iron deficiency anemia is the most common human nutritional disorder, affecting 32.9% of people worldwide. Zinc deficiency affects 17% of the world’s population, with the highest risk occurring in sub-Saharan Africa and South Asia [53,54]. The relatively higher levels of iron and zinc in the advanced lines compared to the other common cereals could contribute to a better daily intake of these important minerals. It is important to emphasize that quality traits related to mineral and protein content in cereals are quantitative traits controlled by many genes. However, the fact that *A*. *magna* is in the secondary gene pool of *A*. *sativa* justifies its domestication to capture its high protein and mineral content without producing infertile hybrids [42].

The effect of the growing site on mineral content was obviously significant (see Table 4), with the Alnif site being significantly higher than the other two sites for five of the ten minerals tested (K, P, Mg, Cu, Mn) and at statistical parity with the highest abundance sites for two other minerals (Fe and S). The El Kbab site produced groats with the second highest mineral content, had the highest Zn values, and was at parity with Alnif for Fe. Bouchane generally had lower mineral contents than Alnif and El Kbab, except for Ca, where it was substantially the highest. According to Doehlert et al. [55], variation in macronutrients is usually due to their availability in the soil. The soil in Bouchane usually has lower fertility due to its sandy nature, coupled with overgrazing [56].

### 3.3. Multivariate Analysis

#### 3.3.1. Correlations

Multivariate analysis (correlation and PCA) was used to identify the relationships between the chemical components and to highlight the similarities of the advanced lines by grouping them in clusters. To obtain genetic improvements in oats, the correlation between traits is one of the key parameters used to guide indirect selection [57,58]. Hawerroth et al. [59] suggested that it is important to evaluate oat genotypes from a large number of environments to identify more stable relationships across growing conditions. The correlation and PCA results from the very different sites selected for this study should help oat breeders to make selection decisions. Correlation values between chemical elements are shown in Table 6.

For macronutrients, the results show that protein is negatively correlated with fat (−0.764) and starch (−0.517), and positively correlated with ash (0.451). These variations could lead to different end uses for these advanced lines, since *A*. *sativa* genotypes used for human consumption have historically been selected for their lower lipid content—resulting in greater stability and fewer calories—as well as their high protein and dietary fiber (especially β-glucan) content [60,61,62]. In contrast, for use as animal feed, it is desirable to select oat genotypes with high lipid and protein content to promote weight gain [63,64,65]. In this case, the relationship between protein and fat content in oat caryopsis is crucial. The different scenarios of correlation between protein and fat in oats have been investigated in previous studies, suggesting that growing conditions can significantly affect the relationship [61,64,66]. This is important for oat breeders to consider when making selection decisions.

The second strongest correlation found among the macronutrients in this study is the positive correlation between fat and fiber (0.686). Dietary fiber is much more abundant in the outer layers of cereal grains, while more than 50% of the lipids are concentrated in the inner starchy endosperm. The outer grain coat consists of bran layers (pericarp, coat seed, and aleurone), which mainly represent the insoluble dietary fiber fraction of the grain [67]. Explaining the correlation between fat and fiber is complicated by the fact that both the fat and fiber contents of the grain are both altered by the dehulling process, in which part of the bran layer is removed along with the hull [68]. Consequently, the fat and fiber values for dehulled groats reported in Table 2 represent the composition after processing, rather than as harvested. Hawerroth et al. [59] also reported a positive correlation between total dietary fiber and fat while evaluating the relationship between chemical constituents of oat caryopsis genotypes (*A*. *sativa*) but did not mention a specific cause.

Much of the protein and starch in oats is found in the endosperm, while the germ contains mainly lipids and protein. According to the correlation results, the more starch a line accumulates, the lower its protein content. This negative correlation was also mentioned by Hawerroth et al. [59].

Most minerals showed significant correlations (both positive and negative) with protein, fiber, and, to a greater extent, fat. Sodium did not correlate significantly with other macro- and micronutrients, while zinc had the most significant correlations. This lack of correlation with sodium may be related to its lower concentration in *A*. *magna* species [30]. Copper had a significant and positive correlation with iron (0.480), manganese (0.472), and zinc (0.360), but no other significant association was found among the microelements. Among the macroelements, phosphorus, potassium, and magnesium were positively correlated with each other with the highest magnitudes (0.883; 0.763; and 0.756). Calcium is a unique phenomenon, with positive correlation coefficients for relationships with fat and fiber, while the other eight minerals (except sodium) had negative coefficients for these macronutrients and each other. Calcium was also negatively correlated with protein, while all other minerals (except sodium) had positive coefficients with protein. Calcium has been shown to be an important metal ion that competes with other minerals for chelation and transport of elements [69]. For example, Brown [69] showed that the addition of calcium carbonate corrected manganese toxicity symptoms in soybeans and was less readily absorbed by iron-efficient oat genotypes.

Calcium is positively correlated with fat (0.801) and fiber (0.764), which in turn are negatively correlated with phosphorus (−0.733 and −0.513, respectively) and zinc (−0.738 and −0.593, respectively). Therefore, calcium, phosphorus, and zinc could play a role in fat and fiber biosynthesis in the advanced lines of *A*. *magna* or vice versa. The significant correlations between fat and all the micronutrients, except sodium and manganese, suggest the possible involvement of these minerals in the different and complex pathways of fatty acid synthesis. Fat has positive correlations among the macroelements, with only sodium and calcium. Phosphorus, sulfur, zinc, and calcium are the nutrients that most influence the protein content (0.549, 0.712, 0.718, and −0.636, respectively). Since calcium is an important component of plant cell walls [70], which are high in fiber and low in protein, it seems logical that calcium would be negatively correlated with protein and positively correlated with fiber (0.764). Zinc, in turn, is an important structural, enzymatic, and regulatory component of many proteins and enzymes [71]; this may account for its positive correlation with protein content in the *A*. *magna* lines. Zinc is also a mineral that interacts remarkably with several chemical components, both macronutrients and micronutrients, with magnitudes greater than 0.500. Thus, the correlations in Table 6 could generally indicate that *A*. *magna* lines with higher zinc content tend to have relatively higher protein and other micronutrients on the one hand, and relatively lower fat, fiber, starch, and calcium content on the other.

The effect of dehulling, mentioned earlier in relation to fat and fiber correlation, could also affect mineral correlation results. Some previous studies have examined the effects of grain pearling on the distribution of minerals in grains, particularly in wheat. Pearling is the use of abrasion and friction to remove the outer bran layers from cereal grains, leaving the more nutritious aleurone layer largely intact in the kernel [72]. In a study on wheat, De Brier et al. [73] showed that xylem mobile elements (Mn, Si, Ca, and Sr) are predominant in the outermost bran layer, whereas phloem mobile elements (K, Mg, P, Fe, Zn, and Cu) are more concentrated in the aleurone. A similar distribution in oats could explain the inverse relationship between the concentration of calcium and those of phosphorus, potassium, and magnesium since the oat grains were dehulled before analysis. Plant uptake of soil minerals depends on the composition of the soil solution but is not a simple translocation. Plants confronted with soil mineral deficiencies, excesses, or imbalances have different ways of transferring minerals from the roots to the grain, and mineral uptake is influenced by a number of mechanisms. These include activation of specific root ion channels; microbial or chemical solubilization enabled by the specific chemistry of the rhizosphere, the relative root/shoot growth ratio, and trace element binding induced by chelation, precipitation, or compartmental storage processes to protect against toxicity [74]. Thus, the increased levels of iron and zinc in these advanced *A*. *magna* lines may be due to a specific ability to translocate more zinc and iron than common oat (*A*. *sativa*) through one or more of these adaptations. Another aspect that may influence the relationship between micronutrients is phytate, the major storage form of phosphorus in cereal grains. Phosphorus bound to inositol, mainly as phytate, represents between 60 and 80% of the total phosphorus in cereals. The phytate binding of trace elements in plants could explain the significant and positive correlations observed in this study between the concentration of phosphorus and those of magnesium (0.756), zinc (0.679), and copper (0.606). This hypothesis is consistent with De Brier et al. [73], who suggested that the correlations between phosphorus and the minerals magnesium, zinc, copper, and iron are related to their association with phytate. Phytate is known to strongly chelate divalent cations such as calcium, iron, and zinc, leading to antinutritional effects in humans and monogastric animals and contributing to mineral deficiencies [75].

#### 3.3.2. Principal Component Analysis

**Figure 1:**
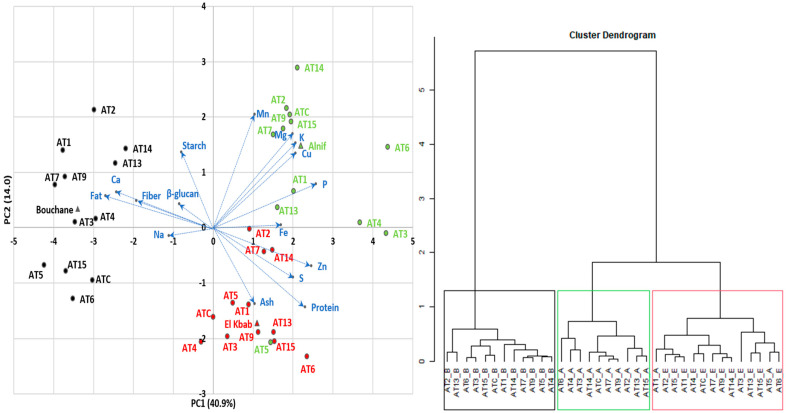
Results of PCA and Dendrogram Analysis (Colors represent the lines from a particular growing site; Green: Alnif; Black: Bouchane; Red: El Kbab; Blue represents the chemical components. Blue arrows represent the the discrimination magnitude between the *A. magna* ssp. *domestica* lines attributed to each chemical component.

The results of the PCA and dendrogram analysis are shown in Figure 1. The first axis of the biplot (PC1) is positively explained by phosphorus (11.3%), zinc (10.3%), and sulfur (6.76%) content and negatively explained by fat (12.4%), calcium (9.96%), and dietary fiber (6.28%) content. The second axis (PC2) is positively related to manganese (20.9%), magnesium (14.3%), starch (9.22%), potassium (11.6%), and copper (9.03%), and negatively related to protein (10.2%) and ash (9.40%). Furthermore, 10.1% of the variability was attributed to the third axis, which was more explained by β-glucan (32.1%). However, in this evaluation, β-glucan, sodium, and iron contribute less to the discrimination between the *A*. *magna* lines, meaning that the magnitude of the line length for these components is low. β-glucan and iron levels were not even significantly different between lines (see Table 2 and Table 4). Although β-glucan is not a point of PCA discrimination, and the advanced lines were lower in β-glucan than typical levels in *A*. *sativa* and barley, their levels exceeded those of common cereals, as mentioned previously. According to Tiwari and Cummins [76], using a Monte Carlo simulation technique, genotype selection was an effective method for improving the final β-glucan level in oats. The other input parameters were environmental conditions (location, soil types, precipitation, and temperature), agronomic practices (sowing date, fertilization, irrigation, harvest), and storage conditions (duration and temperature). Of course, if there is no genetic variation for β-glucan content, genotypic selection for improving this trait will not be effective, at least unless a wide crossing program is initiated with other species like *A*. *sativa* [47]. The other genetically close CCDD (formerly AACC) species are likewise known to be relatively low in β-glucan content [25].

Based on the relationship between the PCA components and the traits, and the support of the dendrogram, three groups were highlighted for their similarities. Growing conditions first determine the distribution of the lines, as shown in Figure 1. When lines are grown at the Bouchane site (black color), the kernels tend to contain more fat, fiber, and calcium and relatively less phosphorus and zinc. This was more pronounced in AT1, AT2, AT7, and AT9. The lines from the Alnif area (green color) were divided into a group with high levels of phosphorus, zinc, sulfur, and iron (AT3, AT4, AT6) and another cluster with relatively high levels of starch, manganese, magnesium, potassium, and copper (AT2, AT7, AT9, AT14, AT15, and ATC). The El Kbab locality (red color) is characterized by high protein and ash content and relatively low starch content, probably due to an offsetting compensation between protein and starch, as commonly reported in cereals. This group is mainly represented by lines AT3, AT6, AT13, AT15. Alnif’s AT5 (in green) behaved in the same way as the latter group.

The overall geographic grouping pa””ern ’n the PCA plot suggests some degree of environmental adaptation of the lines, which could guide their use according to a specific set of desirable traits. It is worth noting that there is a small degree of overlap between these groups, which increases the difficulty of selecting lines for specific compositional goals. Hawerroth et al. [59], working with several Brazilian oat (*A*. *sativa*) cultivars, encountered similar challenges in associating genetic lines with caryopsis compositional quality across multiple phenotypic traits. However, in the absence of a relationship between two traits, genetic improvement of one trait can be achieved without affecting the second one.

#### 3.3.3. Analysis of Protein, Fat, and Fiber Stability of 12 Lines of *A. magna domestica* ssp.

AMMI analysis can be used to identify genotype-by-environment interactions in cereals grown at different locations. It is typically performed on protein, fat, and fiber content, as these are the key traits that guide the selection to the end use of the product, whether for human consumption or animal feed. The objective of this test was to try to understand which traits are more influenced by the growing conditions and how the advanced lines interact with different environments.

The data presented in Table 7 show that the differences in fat content of the advanced line groats are mainly explained by the growing conditions, with the location explaining 81% of the variation in this variable. This was also the case for fiber content, where site location explained 65% of the variation. These results could be explained by the data presented previously, where it was shown that fat content of these advanced lines is strongly correlated with several minerals (Ca, P, S, Zn, Fe, etc.). The potential mobilization and assimilation of nutrients from root to grain could be related to soil fertility and water availability. Contrary to our results, Zhou et al. [77] studied different Australian genotypes of *A*. *sativa* and found that the varietal effect on lipid concentration and composition was predominant. However, Givens et al. [16] reported a strong dependence of oat lipid content on meteorological conditions, which Zhou et al. [77] suggested may be enhanced by low growing temperatures. The effect of different locations on protein and lipid content could be influenced by the environmental conditions during the growth cycle stage of their biosynthesis. In fact, according to Banás et al. [78], most of the lipid stored in the caryopsis of oat (*A*. *sativa*) is deposited during the first half of the grain development stage when the endosperm is still liquefied, whereas both protein and starch deposition occur at the same rate during late grain development. In this study, samples from Bouchane had the highest fat content (9.78%). At this site, droughts are very frequent during the growing season; only 250.2 mm of rainfall was recorded. The limitation of water availability could favor fat biosynthesis. For example, in maize and wheat, oleic and palmitic acid contents are reported to increase under water stress, while linoleic acid decreases [79,80]. Fischer et al. [81] also found a significant increase in the fat content of quinoa due to water limitation. Increases in fat content under water stress are more common in oilseed crops such as sunflower, canola, and safflower, etc. Since fiber is positively correlated with fat, it was also higher in Bouchane (8.35%) compared to Alnif (6.84%) and El Kbab (6.49%). In contrast to fat and fiber, a relevant 25% of the variation in protein rate is related to the genetic background of the advanced *A*. *magna* lines, confirming that tetraploid oats are a good reservoir of useful genes controlling quality traits such as protein. Therefore, they should be a great asset to improve the quality of cultivated oat germplasm through hybridization or domestication as achieved by Ladizinsky [25] and Jackson [26]. The genetic (line) effect on the protein content reported here suggests a much higher heritability of the trait than fat and fiber, suggesting the potential for further progress in protein improvement through genetic selection.

**Figure 2:**
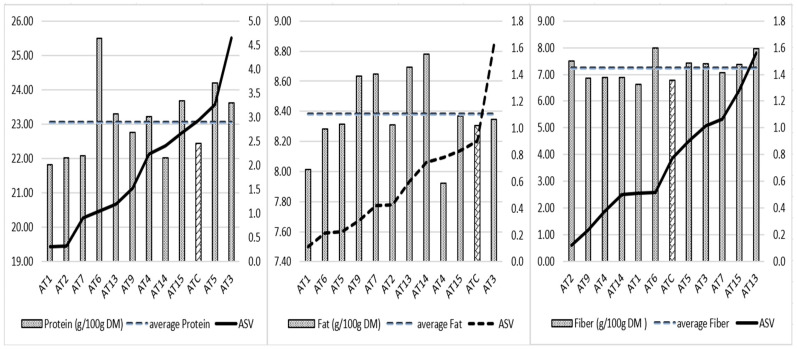
Average protein, fat, and fiber content and AMMI stability values of 12 *A. magna* lines. The bar different to others is the mean protein, fat and fiber of the control ATC respectively.

The protein content and location interaction of 24% is higher than that of fat (14%) and fiber (17%) (Table 7). The AMMI test shows, in Figure 2, the mean values of protein, fat, and fiber of the lines and their stability (ASV). The lower the ASV, the more stable a line’s protein, fat, or fiber content is across the trial locations. Based on protein ASV, advanced lines are grouped into three clusters; cluster 1 (AT1, AT2, AT7, AT6) includes the most stable lines for protein content, with AT6 having the highest protein average of 25.5%. Cluster 2 includes lines AT13, AT9, AT4, AT14, which are characterized by intermediate protein ASV values ranging from 1.2 to 2.4. AT13 and AT4 provide protein contents above the total protein average of the experiment (23.1%). Lines AT15, ATC, AT5, and AT3 form the cluster 3. They have a relatively higher interaction with the environment for the protein content (ASV between 2.7 and 4.6); among them, AT15, AT5, AT3 exceed the mean protein value. In terms of fat content, four lines (AT9, AT7, AT13, AT14) exceeded the overall average fat value of 8.38%. Their fat stability ranged from 0.3 to 0.7. For the fiber content, six of the lines exceeded the mean fiber content of 7.23%; AT2, AT6, and AT13 provided the highest fiber values with good, medium, and low stability (0.1, 0.5, and 1.6, respectively). Considering the three quality components of protein, fat, and fiber (3 degrees) or two of them (2 degrees), only AT13 exceeded the averages of the three quality components. Lines AT3, AT5, AT6, and AT15 have better quality potential for protein–fiber combination.

## 4. Conclusions

The aim of this current work was to carry out the chemical characterization of twelve advanced *A*. *magna* ssp. *domestica* lines grown in three diverse Moroccan sites (semi-desert, montane, Mediterranean). The results show that genetic factors and growing conditions significantly influenced the chemical composition of *A*. *magna domestica* lines, with differences in protein, fat, fiber, starch, and minerals among sites and lines. The mean protein content varied from 20.9% in Bouchane to 24.1% in Alnif, with AT3, AT5, and AT6 lines exhibiting exceptionally high protein content of about 27% in Alnif. High protein, fat, and fiber were observed in several lines compared to the control in the different trial locations. The highest fat content was found in Bouchane (9.78%), while fiber content varied among lines, with AT6 showing the highest mean value of 8.00% across sites. Protein content was negatively correlated with fat and starch and positively correlated with ash. The *A*. *magna domestica* lines showed elevated levels of iron (7.50 × 10^−3^ g/100 g DM) and zinc (3.40 × 10^−3^ g/100 g DM) compared to the common cereals and *A*. *sativa*. This indicates the potential of *A*. *magna* to address micronutrient deficiencies worldwide. In particular, advanced line AT2 had the highest values of calcium, iron, and copper, while AT13 provided the highest values of zinc (3.70 × 10^−3^ g/100 g DM). According to the AMMI stability analysis, fat was more influenced by growing conditions than fiber and protein; 25% of the variability in protein content was attributed to the genetic factor. The ranking of the lines based on the overall mean values of protein, fat, and fiber (23.1; 8.38; 7.23%) shows that only AT13 exceeded the mean values of these macronutrients as well as AT3, AT5, AT6, and AT15 for the protein–fiber combination. This study shows the nutritional potential of the advanced lines compared to the control variety (Avery) registered in the official Moroccan catalog in 2019. For all the chemical components, at least one or more lines exceeded this control. It would be interesting to study the technological characteristics of these lines such as dehulling, milling, and rheological properties in order to further explore their potential applicability in the food industry.

## Figures and Tables

**Table 1:** Location and description of the experimental agro-climatic trial sites.

Experimental Site	Zone	Coordinates		Alt	Soil Type	Temp		Rain	Hum	S.H	Days to Harvest
		Latitude	Longitude			Mn	Mx				
Alnif	Oasis	31°06′37″ N	5°03′50″ W	1320	Fluvisols	9	43	43.2 *	26.2	3529	177
Bouchane	Phosphate Plateau	32°14′35″ N	8°19′45″ W	830	Cambisols	9	38	250.2	54.1	3304	219
El Kbab	Middle Atlas	32°42′25″ N	5°31′57″ W	1540	Fluvisols	4	38	573.0	50.9	3477	206

Alt: altitude (m); Temp: temperature (°C); Rain: total rainfall (mm); Hum: average humidity (%); S.H: total sunshine hours (h); * With an additional 200 m^3^/ha of irrigation during the crop cycle. Climate data were provided by worldweatheronline.com (accessed on 10 April 2023). Reported climate data are averages or totals for the experimental period (between October and July).

**Table 2:** Chemical composition of 12 *A. magna domestica* ssp. lines in g/100 g on dry weight basis (DM).

Line/Site	Protein	Moisture	Fat	Fiber	β-Glucan	Ash	Starch
ATC	22.4 ^f^ ± 1.40	12.9 ^a^ ± 0.14	8.31 ^b^ ± 0.42	6.77 ^de^ ± 0.51	2.49 ^a^ ± 0.07	2.49 ^b^ ± 0.03	57.0 ^b^ ± 1.28
AT1	21.8 ^g^ ± 1.82	12.7 ^ab^ ± 0.15	8.01 ^c^ ± 1.05	6.62 ^e^ ± 0.69	2.77 ^a^ ± 0.20	2.60 ^ab^ ± 0.05	58.2 ^a^ ± 1.53
AT2	22.0 ^fg^ ± 1.90	12.8 ^ab^ ± 0.36	8.31 ^b^ ± 1.26	7.51 ^b^ ± 0.96	2.74 ^a^ ± 0.38	2.5 ^b^ ± 0.19	58.1 ^a^ ± 1.13
AT3	23.6 ^c^ ± 2.80	12.8 ^ab^ ± 0.51	8.35 ^b^ ± 1.41	7.40 ^bc^ ± 1.44	2.69 ^a^ ± 0.43	2.72 ^a^ ± 0.25	55.7 ^c^ ± 0.95
AT4	23.2 ^d^ ± 2.23	12.7 ^b^ ± 0.18	7.92 ^c^ ± 1.62	6.87 ^de^ ± 1.09	2.34 ^a^ ± 0.15	2.51 ^b^ ± 0.09	57.4 ^ab^ ± 1.02
AT5	24.2 ^b^ ± 2.44	12.6 ^b^ ± 0.18	8.31 ^b^ ± 1.10	7.41 ^bc^ ± 0.71	2.42 ^a^ ± 0.11	2.59 ^ab^ ± 0.15	57.0 ^b^ ± 1.04
AT6	25.4 ^a^ ± 2.05	12.6 ^b^ ± 0.22	8.28 ^b^ ± 1.26	8.00 ^a^ ± 1.06	2.60 ^a^ ± 0.05	2.58 ^ab^ ± 0.22	54.9 ^d^ ± 0.45
AT7	22.0 ^fg^ ± 2.14	12.9 ^a^ ± 0.23	8.65 ^a^ ± 1.21	7.07 ^cd^ ± 0.62	2.42 ^a^ ± 0.19	2.44 ^b^ ± 0.09	58.1 ^a^ ± 0.70
AT9	22.8 ^e^ ± 1.45	12.8 ^ab^ ± 0.28	8.63 ^a^ ± 1.05	6.85 ^de^ ± 0.81	2.45 ^a^ ± 0.26	2.52 ^b^ ± 0.11	57.7 ^ab^ ± 0.67
AT13	23.3 ^d^ ± 2.63	12.7 ^ab^ ± 0.21	8.69 ^a^ ± 0.79	7.96 ^a^ ± 1.50	2.63 ^a^ ± 0.39	2.62 ^ab^ ± 0.10	57.1 ^b^ ± 0.91
AT14	22.0 ^fg^ ± 0.53	12.8 ^ab^ ± 0.32	8.78 ^a^ ± 1.17	6.88 ^de^ ± 0.74	2.20 ^a^ ± 0.02	2.42 ^b^ ± 0.17	58.0 ^a^ ± 1.11
AT15	23.7 ^c^ ± 1.71	12.7 ^ab^ ± 0.28	8.37 ^b^ ± 1.36	7.38 ^bc^ ± 1.20	2.64 ^a^ ± 0.21	2.56 ^ab^ ± 0.16	57.5 ^ab^ ± 0.73
Alnif	24.1 ^a^ ± 2.05	12.8 ^a^ ± 0.31	7.56 ^c^ ± 0.60	6.84 ^b^ ± 0.57	2.52 ^a^ ± 0.29	2.57 ^a^ ± 0.16	57.2 ^b^ ± 1.22
Bouchane	20.9 ^b^ ± 1.11	12.6 ^b^ ± 0.20	9.78 ^a^ ± 0.40	8.35 ^a^ ± 0.84	2.61 ^a^ ± 0.22	2.49 ^b^ ± 0.12	57.7 ^a^ ± 1.46
El Kbab	24.2 ^a^ ± 1.21	12.8 ^a^ ± 0.27	7.82 ^b^ ± 0.48	6.49 ^c^ ± 0.50	2.46 ^a^ ± 0.27	2.59 ^a^ ± 0.19	56.9 ^b^ ± 1.35

Note: Results are from ANOVA 2. Values within a column with the same superscript letters (a, b, c, d, e, f, g) indicate that the difference between the means is not statistically significant at the 0.05 level and the lines belong to the same homogenous group based on Newman–Keuls post hoc test. Last three rows indicate means at each site.

**Table 3:** Chemical composition of the cereal grains [40] compared to *A. magna* in DM (g/100 g).

	Wheat	Rye	Corn	Barley	Rice	Millet	*A. sativa*	*A. magna* *
Moisture	12.6	13.6	11.3	12.1	13.0	12.0	13.1	12.8 (12.3–13.6)
Protein (*N* × 6.25)	11.3	9.4	8.8	11.1	7.7	10.5	10.8	23.1 (19.4–27.4)
Lipids	1.8	1.7	3.8	2.1	2.2	3.9	7.2	8.4 (6.4–10.3)
Available carbohydrates	59.4	60.3	65.0	62.7	73.7	68.2	56.2	57.2 ** (54.0–61.1)
Fiber	13.2	13.1	9.8	9.7	2.2	3.8	9.8	7.2 (5.6–10.2)
Minerals	1.7	1.9	1.3	2.3	1.2	1.6	2.9	2.6 (2.1–3.1)

* Values of *A. magna* lines from this study. Min, Max in parentheses. ** the starch value is reported here.

**Table 4:** Mineral composition of the 12 *A. magna* ssp. *domestica* advanced lines on a dry matter (DM) basis.

	Macroelements (×10^−1^ g/100 g DM Basis)						Microelements (×10^−3^ g/100 g DM Basis)			
Line/Site	Na	K	Ca	P	S	Mg	Fe	Zn	Cu	Mn
ATC	0.03 ^b^ ± 0.00	3.65 ^a^ ± 0.04	0.65 ^c^ ± 0.01	4.65 ^ab^ ± 0.05	2.08 ^c^ ± 0.02	1.44 ^c^ ± 0.02	6.80 ^a^ ± 0.97	3.30 ^ab^ ± 0.66	0.55 ^ab^ ± 0.16	4.26 ^cd^ ± 0.58
AT1	0.03 ^b^ ± 0.01	3.38 ^ab^ ± 0.02	0.71 ^abc^ ± 0.01	4.52 ^ab^ ± 0.02	2.12 ^c^ ± 0.02	1.46 ^bc^ ± 0.01	8.22 ^a^ ± 2.57	3.27 ^ab^ ± 0.41	0.57 ^ab^ ± 0.15	4.46 ^abcd^ ± 0.14
AT2	0.03 ^ab^ ± 0.00	3.55 ^ab^ ± 0.05	0.77 ^a^ ± 0.01	4.66 ^ab^ ± 0.04	2.23 ^abc^ ± 0.01	1.60 ^ab^ ± 0.01	9.33 ^a^ ± 3.10	3.16 ^b^ ± 0.30	0.59 ^a^ ± 0.13	4.80 ^ab^ ± 0.07
AT3	0.04 ^a^ ± 0.00	3.68 ^a^ ± 0.05	0.68 ^bc^ ± 0.01	4.67 ^ab^ ± 0.06	2.23 ^abc^ ± 0.02	1.53 ^abc^ ± 0.01	7.70 ^a^ ± 1.57	3.32 ^ab^ ± 0.61	0.55 ^ab^ ± 0.13	4.61 ^abcd^ ± 0.50
AT4	0.04 ^ab^ ± 0.00	3.48 ^ab^ ± 0.07	0.69 ^abc^ ± 0.01	4.53 ^ab^ ± 0.07	2.32 ^ab^ ± 0.01	1.52 ^abc^ ± 0.02	8.04 ^a^ ± 2.59	3.32 ^ab^ ± 0.50	0.49 ^b^ ± 0.15	4.34 ^bcd^ ± 0.28
AT5	0.04 ^a^ ± 0.00	3.03 ^b^ ± 0.04	0.72 ^abc^ ± 0.01	4.26 ^b^ ± 0.06	2.22 ^abc^ ± 0.02	1.48 ^bc^ ± 0.01	7.60 ^a^ ± 1.92	3.30 ^ab^ ± 0.48	0.49 ^b^ ± 0.05	4.23 ^d^ ± 0.29
AT6	0.03 ^b^ ± 0.00	3.67 ^a^ ± 0.06	0.74 ^ab^ ± 0.01	4.76 ^a^ ± 0.09	2.35 ^a^ ± 0.02	1.57 ^abc^ ± 0.01	8.00 ^a^ ± 1.74	3.52 ^ab^ ± 0.65	0.58 ^a^ ± 0.20	4.50 ^abcd^ ± 0.64
AT7	0.03 ^ab^ ± 0.00	3.45 ^ab^ ± 0.03	0.72 ^abc^ ± 0.01	4.61 ^ab^ ± 0.06	2.24 ^abc^ ± 0.02	1.52 ^abc^ ± 0.01	7.18 ^a^ ± 0.83	3.63 ^a^ ± 0.79	0.50 ^b^ ± 0.10	4.79 ^ab^ ± 0.61
AT9	0.03 ^ab^ ± 0.00	3.45 ^ab^ ± 0.04	0.70 ^abc^ ± 0.01	4.54 ^ab^ ± 0.04	2.20 ^abc^ ± 0.02	1.53 ^abc^ ± 0.01	7.24 ^a^ ± 1.22	3.45 ^ab^ ± 0.68	0.53 ^ab^ ± 0.10	4.70 ^abc^ ± 0.43
AT13	0.03 ^b^ ± 0.00	3.55 ^ab^ ± 0.06	0.74 ^ab^ ± 0.01	4.51 ^ab^ ± 0.05	2.40 ^a^ ± 0.02	1.60 ^ab^ ± 0.00	6.53 ^a^ ± 0.66	3.70 ^a^ ± 0.69	0.57 ^ab^ ± 0.11	4.49 ^abcd^ ± 0.23
AT14	0.04 ^ab^ ± 0.00	3.62 ^a^ ± 0.05	0.65 ^c^ ± 0.01	4.70 ^a^ ± 0.04	2.25 ^abc^ ± 0.01	1.63 ^a^ ± 0.01	7.10 ^a^ ± 1.05	3.66 ^a^ ± 0.57	0.56 ^ab^ ± 0.08	4.90 ^a^ ± 0.45
AT15	0.04 ^ab^ ± 0.00	3.68 ^a^ ± 0.05	0.70 ^abc^ ± 0.01	4.57 ^ab^ ± 0.05	2.26 ^abc^ ± 0.01	1.49 ^bc^ ± 0.02	6.42 ^a^ ± 1.04	3.68 ^a^ ± 0.84	0.55 ^ab^ ± 0.09	4.40 ^bcd^ ± 0.51
Alnif	0.03 ^b^ ± 0.00	4.01 ^a^ ± 0.03	0.64 ^b^ ± 0.01	5.09 ^a^ ± 0.03	2.27 ^a^ ± 0.01	1.64 ^a^ ± 0.01	8.00 ^a^ ± 2.34	3.70 ^b^ ± 0.25	0.67 ^a^ ± 0.10	4.80 ^a^ ± 0.54
Bouchane	0.04 ^a^ ± 0.00	3.24 ^b^ ± 0.02	0.84 ^a^ ± 0.01	4.09 ^c^ ± 0.28	2.12 ^b^ ± 0.01	1.46 ^b^ ± 0.01	6.47 ^b^ ± 0.80	2.80 ^c^ ± 0.16	0.47 ^b^ ± 0.04	4.39 ^b^ ± 0.41
El Kbab	0.03 ^b^ ± 0.00	3.29 ^b^ ± 0.03	0.65 ^b^ ± 0.01	4.57 ^b^ ± 0.04	2.34 ^a^ ± 0.02	1.49 ^b^ ± 0.01	8.06 ^a^ ± 1.51	3.86 ^a^ ± 0.54	0.50 ^b^ ± 0.10	4.40 ^b^ ± 0.23

Note: Results are from ANOVA 2. Values within a column with the same superscript letters (a, b, c, d) indicate that the difference between the means is not statistically significant at the 0.05 level and the lines belong to the same homogenous group based on Newman–Keuls post hoc test. Macroelements are Na, K, Ca, P, S, Mg. Microelements are Fe, Zn, Cu, Mn.

**Table 5:** Chemical composition of cereal grains [52] compared to *A. magna* (in ×10^−1^ g/100 g DM basis for macroelements and ×10^−3^ g/100 g DM basis for microelements).

	Wheat	Rye	Corn	Barley	Rice	Millet	*A. sativa*	*A. magna* *		
	Mean							Mean	Min	Max
Sodium	0.02	0.02	0.35	0.12	0.05	0.05	0.02	0.03	0.02	0.04
Potassium	4.94	5.10	2.87	4.52	0.92	1.95	4.29	3.53	2.70	4.70
Calcium	0.30	0.24	0.15	0.33	0.24	0.08	0.54	0.70	0.50	1.00
Phosphorous	3.04	3.32	2.56	2.64	0.94	2.85	5.23	4.57	3.50	5.80
Magnesium	1.60	1.10	1.20	1.33	0.20	1.14	1.77	1.53	1.30	1.80
Iron	3.30	2.60	2.40	3.60	0.80	3.00	4.70	7.50	4.50	14.0
Zinc	2.90	2.70	2.20	2.80	1.30	1.70	4.00	3.40	2.50	4.80
Copper	0.40	0.40	0.30	0.50	0.20	0.80	0.60	0.50	0.30	0.90
Manganese	4.00	2.60	0.50	1.90	1.10	1.60	4.90	4.50	3.50	5.80

* Mean, min, and max values of the *A. magna* lines from this study. Macroelements are Na, K, Ca, P, S, Mg. Microelements are Fe, Zn, Cu, Mn.

**Table 6:** Correlation coefficients and their significance between chemical elements of the twelve *A. magna* ssp. *domestica* lines.

	Fat	Fiber	β-Glucan	Starch	Ash	Na	K	Ca	P	S	Mg	Fe	Zn	Cu	Mn
Protein	−0.764 **	−0.379 *	−0.324	−0.517 **	0.451 **	−0.141	0.349 *	−0.636 **	0.549 **	0.712 **	0.276	0.324	0.718 **	0.357 *	−0.041
Fat	1	0.686 **	0.277	0.227	−0.326	0.311	−0.532 **	0.801 **	−0.733 **	−0.539 **	−0.403 *	−0.583 **	−0.738 **	−0.515 **	−0.132
Fiber		1	0.131	−0.048	−0.153	0.159	−0.240	0.764 **	−0.513 **	−0.331 *	−0.191	−0.407 *	−0.593 **	−0.277	−0.113
Ash			1	−0.121	0.243	0.059	0.023	0.397 *	−0.100	−0.313	−0.068	−0.119	−0.376 *	−0.056	−0.102
Starch				1	−0.413 *	0.135	−0.105	0.180	−0.146	−0.237	−0.059	−0.234	−0.110	−0.054	0.194
β-glucan					1	−0.074	0.097	−0.311	0.215	0.253	0.075	0.174	0.302	0.143	−0.170
Na						1	−0.152	0.297	−0.233	−0.231	−0.204	−0.386 *	−0.235	−0.263	−0.189
K							1	−0.378 *	0.883 **	0.206	0.763 **	0.233	0.408 *	0.720 **	0.382 *
Ca								1	−0.556 **	−0.442 **	−0.312	−0.411 *	−0.690 **	−0.471 **	−0.198
P									1	0.431 **	0.756 **	0.342 *	0.679 **	0.606 **	0.338 *
S										1	0.381 *	0.277	0.745 **	0.253	0.135
Mg											1	0.334 *	0.418 *	0.633 **	0.581 **
Fe												1	0.242	0.480 **	0.218
Zn													1	0.360 *	0.156
Cu														1	0.472 **

* <5% level and ** <1% level of probability.

**Table 7:** AMMI analysis of variance for protein, fat, and fiber of 12 *A. magna* ssp. *domestica* lines.

		Protein			Fat			Fiber		
Source	Df	Sum Sq	F Value	Var ^a^	Sum Sq	F Value	Var ^a^	Sum Sq	F Value	Var ^a^
Site (E)	2	244.07	502.67 ***	51%	105.74	1722.84 ***	81%	70.52	496.95 ***	65%
Rep (Site)	6	1.46	13.39	-	0.18	5.15	-	0.43	0.84	-
Line (G)	11	117.79	590.46 ***	25%	6.88	105.06 ***	5%	20.67	22.34 ***	19%
G × E	22	116.84	292.85 ***	24%	18.35	140.10 ***	14%	18.12	9.79 ***	17%
IPCA1	12	90.05	413.77 ***	77%	11.19	156.68 ***	61%	11.87	11.76 ***	66%
IPCA2	10	26.79	147.75 **	23%	7.16	120.20 ***	39%	6.24	7.42 ***	35%
Residuals	66	1.20			0.39			5.55		

**^a^** Var = % of variation explained by each factor (site, line and S × L interaction) as a part of the total sum of squares of the chemical component (protein, fat, fiber). G: Genotype (Line in this case), E: Environment (Site in this case), G × E: Genotype by environment interaction. ***, **: significant at 0.1%, 1% level of probabilty respectively.

## Data Availability

Data are contained within the article.
